# Case Report: Primary splenic hydatid cyst

**DOI:** 10.3389/fmed.2025.1605050

**Published:** 2025-09-12

**Authors:** Bilal Ahmad Wani, Mohammadd Ashraf Khan, Tanish Baweja, Omar Javed Shah, Arushi Sumrani, Tajamul Rashid

**Affiliations:** ^1^Department of Medicine, Hamdard Institute of Medical Sciences and Research, New Delhi, India; ^2^Hamdard Institute of Medical Sciences and Research, New Delhi, India; ^3^Department of Surgery, Hamdard Institute of Medical Sciences and Research, New Delhi, India

**Keywords:** splenic hydatid cyst, *Echinococcus granulosus*, unilocular solitary cyst, zoonotic infection, splenectomy, case report

## Abstract

**Introduction:**

Primary splenic hydatidosis accounts for <2% of global cystic echinococcosis (CE) cases. Its rarity and non-specific clinical presentation often lead to delayed diagnosis, increasing the risk of severe complications. This case is noteworthy because early clinical suspicion, despite subtle symptoms, enabled prompt diagnosis and successful treatment. It highlights the importance of maintaining a broad differential diagnosis when evaluating unexplained abdominal pain, particularly in endemic regions. We present this case along with a focused literature review to raise awareness and offer practical guidance for clinicians encountering similar diagnostic challenges.

**Case presentation:**

A 23-year-old woman presented with chronic pain in the left upper quadrant of her abdomen, accompanied by nausea, vomiting, and low-grade fever. Physical examination revealed splenomegaly. Given her clinical presentation, a splenic hydatid cyst was considered as part of the differential diagnosis. Abdominal ultrasonography (USG) and computed tomography (CT) revealed a solitary cystic lesion in the spleen without calcification or solid components. Serological testing using enzyme immunoassay for IgG (Immunoglobulin G) and IgE (Immunoglobulin E) antibodies was positive for echinococcosis. Due to the large size of the cyst and the risk of rupture, a total splenectomy was performed. Histopathological examination of the resected specimen confirmed the diagnosis of a hydatid cyst. The postoperative course was uneventful, and the patient has continued to do well during regular outpatient follow-ups.

**Conclusion:**

This case highlights that splenic hydatid cysts, although uncommon and often presenting with non-specific symptoms, should be considered in the differential diagnosis of abdominal pain, especially in endemic regions. Early diagnosis through the combined use of imaging and serological tests is essential for effective clinical management. Surgical intervention, based on the cyst’s size and the risk of rupture, can result in excellent outcomes. This case underscores the need for heightened clinical suspicion in endemic regions to mitigate delays in diagnosis.

## Introduction

Hydatid disease, caused by the larvae of *Echinococcus granulosus*, remains a major public health issue, particularly in endemic regions such as the Mediterranean, Australia, New Zealand, North Africa, Eastern Europe, the Balkans, the Middle East, and South America (1, 2). Several states in India report a high prevalence of echinococcosis, with the highest rates observed in Andhra Pradesh, Tamil Nadu, and Kashmir (3).

The liver (70%) and lungs (25%) are the most commonly affected organs by cystic echinococcosis (CE) (4). Splenic involvement in hydatid disease is uncommon, occurring in only 0.5–4% of cases. Primary isolated splenic hydatid cysts (SHCs) are even rarer, constituting less than 2% of all cases, even in regions where the disease is endemic (4, 5). This rarity is primarily due to the filtering action of the liver and lungs, which act as the first and second barriers, preventing most cyst embryos from entering the systemic circulation and reaching organs such as the spleen (6). The spleen’s role as a secondary lymphoid organ with organized immune cell populations may also influence this distribution pattern, although our understanding of the specific mechanisms through which the splenic immune architecture controls different types of infections remains limited (7).

The diagnosis of splenic hydatid disease is often delayed due to its non-specific clinical presentation and overlapping imaging features. Ultrasonography (USG) and computed tomography (CT), while widely used, lack specificity, particularly for non-calcified cysts without daughter cysts, making it difficult to distinguish hydatid cysts from other benign splenic lesions (8). Magnetic resonance imaging (MRI) provides better visualization of complex or atypical cysts but is not routinely used because of its higher cost (1, 8). Serological tests such as enzyme-linked immunosorbent assay (ELISA) and western blot are commonly used for diagnosis, screening, and postoperative follow-up. However, these tests frequently yield inconclusive results, especially in cases involving intact, calcified, or sterile cysts (5). Furthermore, seropositivity rates are higher in hepatic hydatid disease (80–94%) compared to extrahepatic cases (~65%), thereby complicating the diagnostic challenge (9). As a result, many cases are confirmed only intraoperatively or on histopathological examination. If left untreated, hydatid cysts can lead to severe complications such as rupture, secondary infection, fistula formation, and potentially life-threatening anaphylaxis (1).

Managing splenic hydatid disease also presents considerable challenges due to the absence of standardized treatment protocols. Treatment options include total or laparoscopic splenectomy, spleen-preserving surgery, percutaneous aspiration, and medical therapy, each associated with specific risks. Total splenectomy eliminates the chance of recurrence but increases the risk of overwhelming post-splenectomy infection (OPSI), while spleen-preserving techniques may lead to higher recurrence rates (5, 10). Although laparoscopic approaches are less invasive, they carry potential risks such as cyst rupture and anaphylaxis (8).

This report describes a rare case of isolated splenic hydatid disease, detailing the clinical presentation, diagnostic approach, and treatment strategies. By sharing this case, we aim to enhance awareness, promote early recognition, and support evidence-based management of this uncommon yet clinically significant condition.

## Case report

A 23-year-old female homemaker presented with a 2-month history of severe, gradually worsening abdominal pain localized to the left upper quadrant, which radiated to her back. The pain was sharp in nature and accompanied by nausea, vomiting, and loss of appetite. She also experienced persistent low-grade fever, ranging from 99°F to 100°F, without chills, rigors, or rash, which responded to over-the-counter medication. She reported altered bowel habits characterized by loose stools, which began with the onset of pain and resolved within approximately 2 weeks after symptomatic treatment. There was no history of jaundice, constipation, urinary symptoms, blood in stools, or other systemic complaints. She had no significant past medical or surgical history, no known comorbidities, and no family history of genetic disorders. Her diet was predominantly non-vegetarian. She had no known exposure to domestic animals and had been residing in an urban area for the past several years. There was no history of recent travel. Her menstrual cycles were regular, and she had never been pregnant.

On examination, the patient was conscious and oriented to time, place, and person. Her pulse rate was 86 beats per min, blood pressure was 122/82 mmHg, temperature was 98°F, respiratory rate was 16 breaths per min, and SpO2 (Oxygen Saturation) was 99% at room air. Physical examination revealed the presence of splenomegaly.

**Figure 1:**
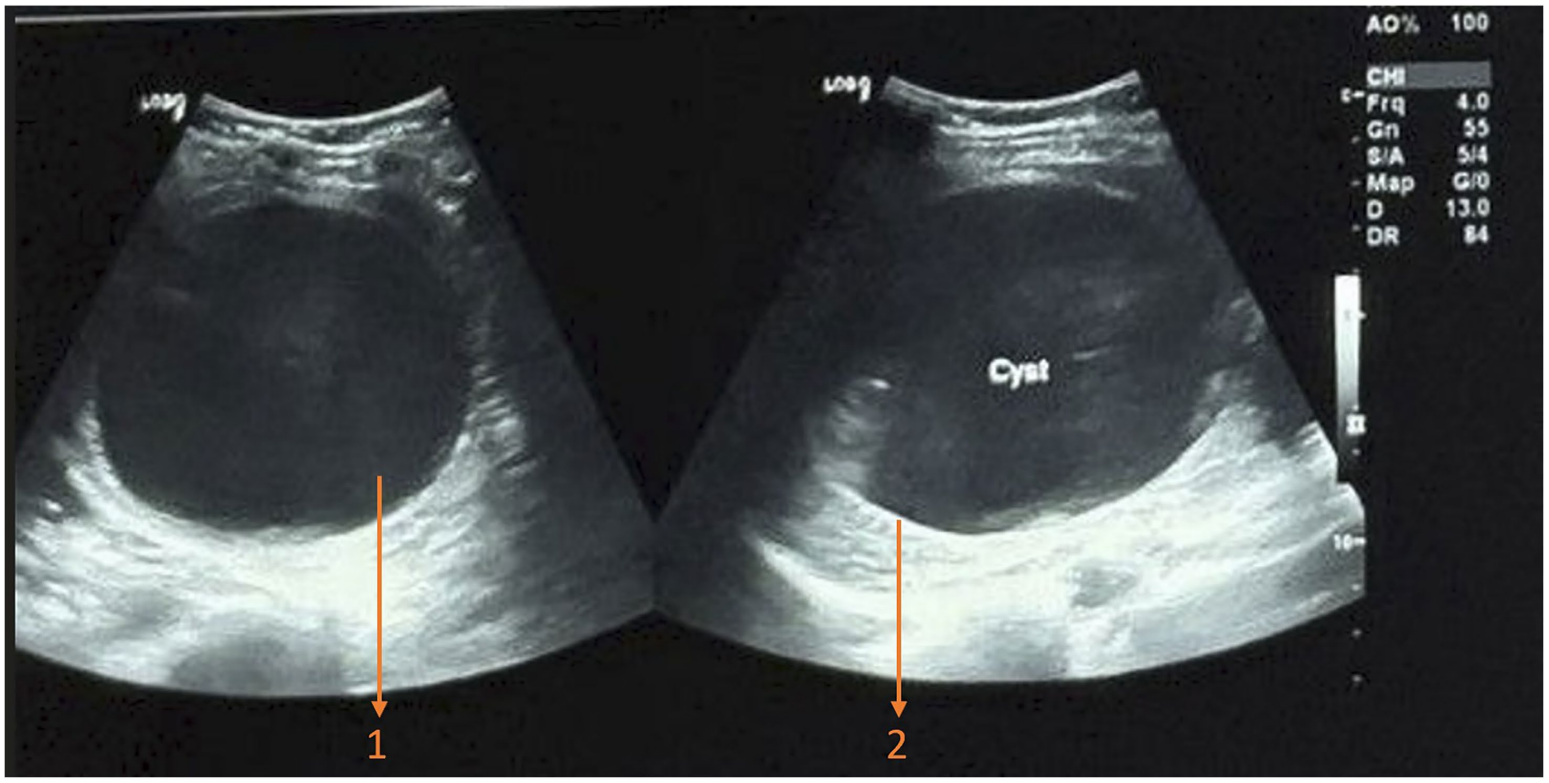
Ultrasound of the whole abdomen showing a solitary anechoic cystic lesion in the spleen. (1) Anechoic cystic cavity with no internal septations. (2) Thickened cyst wall.

**Figure 2:**
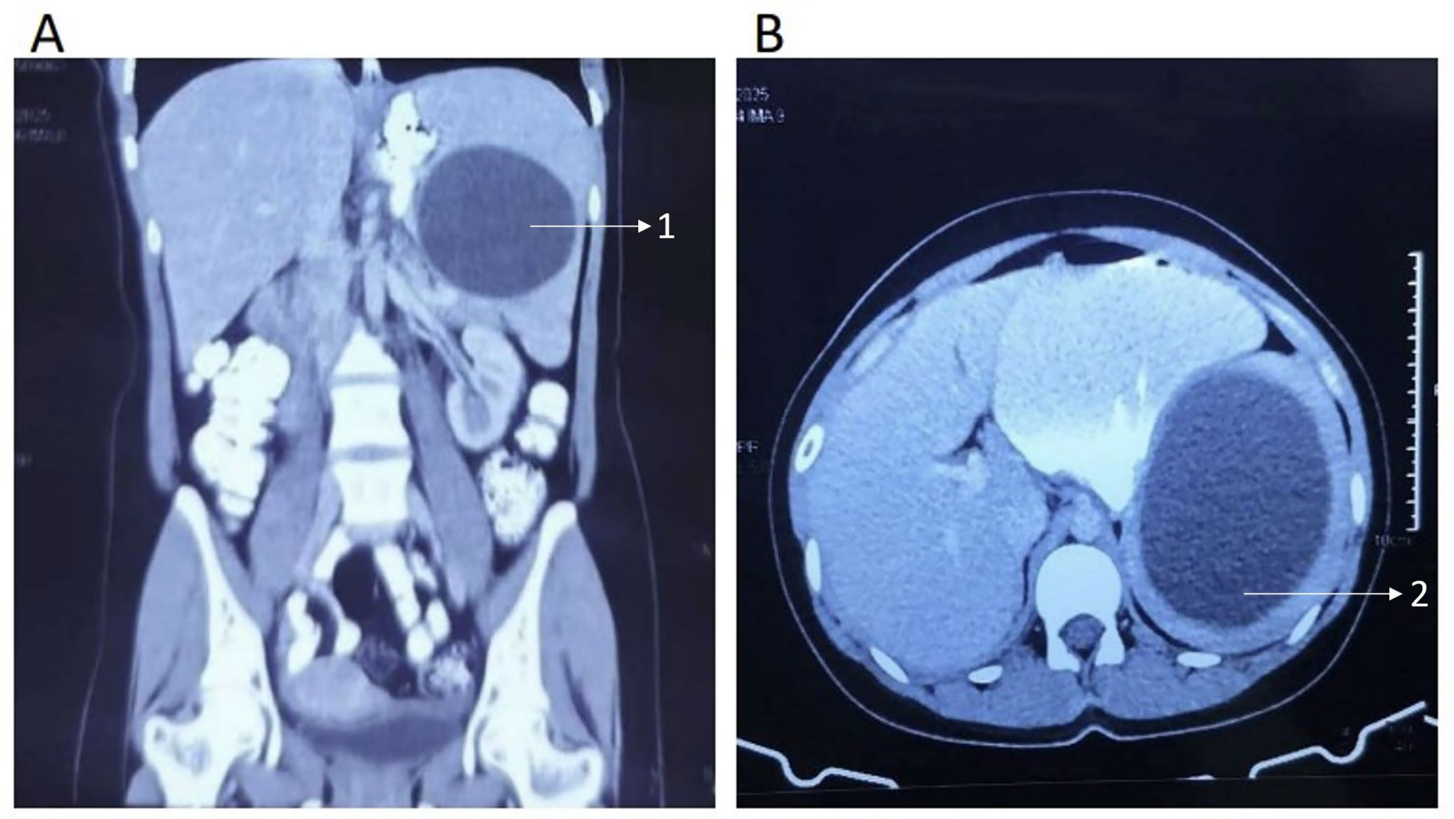
Contrast-enhanced computed tomography (CT) scan showing **(A)** coronal and **(B)** axial sections of the abdomen. A solitary cystic lesion is seen within the spleen, with no evidence of calcification or soft tissue component. (1) Solitary cyst with no internal septations on the coronal section. (2) Solitary cyst with no internal septations on the axial (transverse) section.

Laboratory investigations, including a complete blood count, liver and kidney function tests, random blood sugar, viral markers (human immunodeficiency virus types I and II, hepatitis B and C), and electrocardiogram (ECG), were unremarkable, except for an elevated eosinophil percentage of 10.9% (reference range: 1–6%). Stool examination was normal, effectively ruling out other parasitic infections. Subsequently, radiological investigations were performed. Ultrasonography (USG) of the whole abdomen revealed a spleen with normal shape and echotexture, measuring 13 cm along the long axis. An anechoic cyst measuring 9.7 × 8.7 × 7.9 cm with a wall thickness of 0.4 cm and an estimated volume of 352 cm^3^ was identified within the spleen (Figure 1). Computed tomography (CT) of the abdomen confirmed a large splenic cyst (9.8 × 7.8 cm) without calcification or soft tissue components, along with splenomegaly (14.7 cm in length) (Figure 2). Based on the imaging findings, the lesion was classified as cystic echinococcosis-1 (CE1) according to the World Health Organization Informal Working Group on Echinococcosis (WHO-IWGE) guidelines, consistent with an early or uncomplicated hydatid cyst. No additional cystic lesions were identified in the liver or elsewhere in the abdomen. A chest X-ray showed no evidence of pulmonary involvement. Given the diagnostic clarity provided by USG, CT, and serology, and in view of financial considerations, MRI was not pursued following joint clinical decision-making. Further evaluation with *Echinococcus* serology using enzyme immunoassay revealed an elevated immunoglobulin G (IgG) level of 1.07 (normal: <0.9) and a markedly raised immunoglobulin E (IgE) level of 593.4 IU/mL (normal: <100 IU/mL).

Total open splenectomy under general anesthesia was selected as the treatment approach, given the high risk of rupture and peritoneal spillage in this case. The patient was counseled about the lifelong risk of overwhelming post-splenectomy infection (OPSI), emphasizing the need for continued vaccination and prompt antibiotic use. Before surgery, she was started on a three-week course of albendazole 400 mg twice daily, with her liver function tests (LFTs) remaining within the normal range after the treatment course. In addition, pneumococcal (PPSV23), *Haemophilus influenzae* type b, and meningococcal vaccinations were administered 3 weeks before surgery.

**Figure 3:**
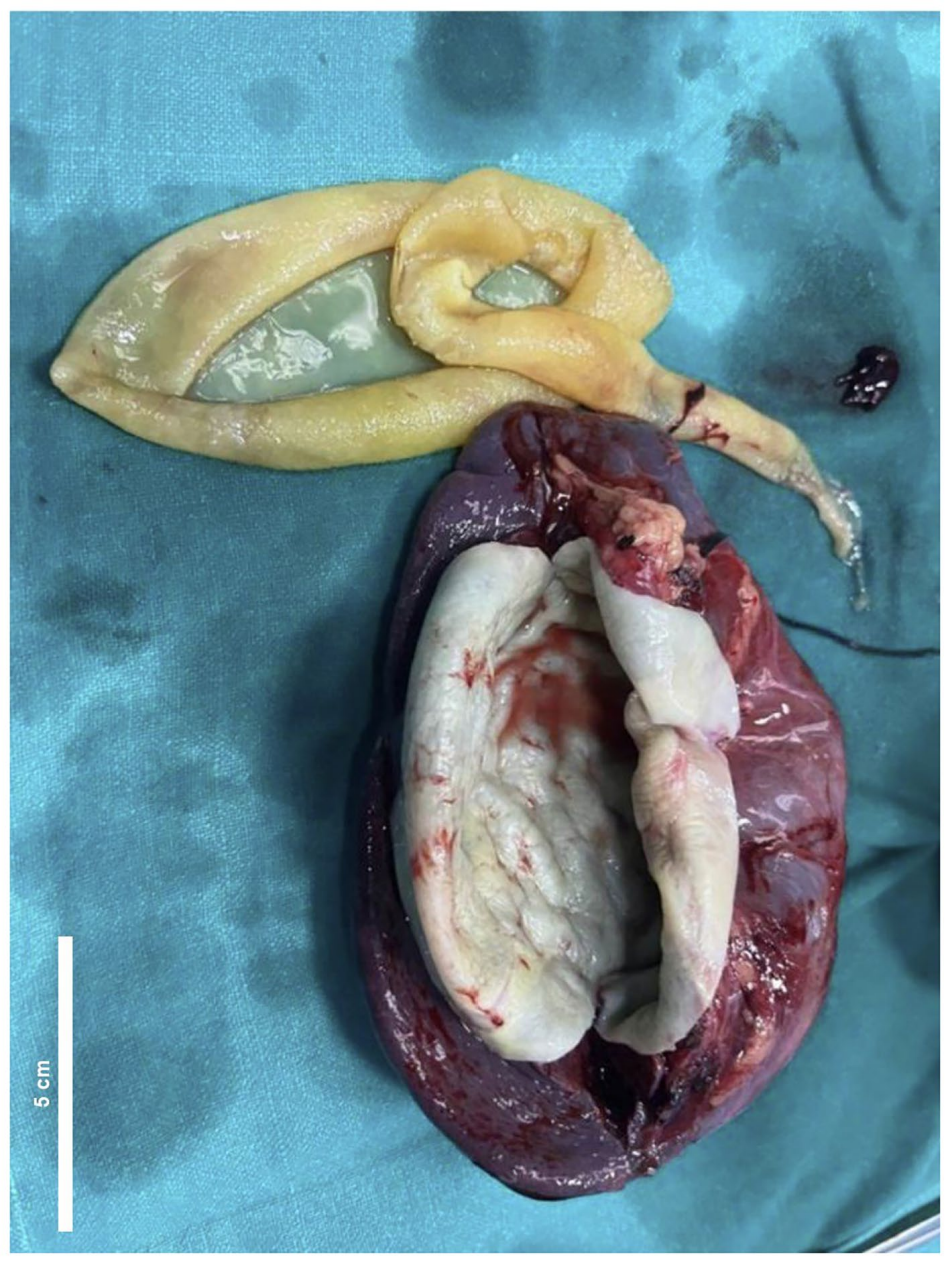
Photo showing the enlarged spleen with a cavity after cyst removal.

**Figure 4:**
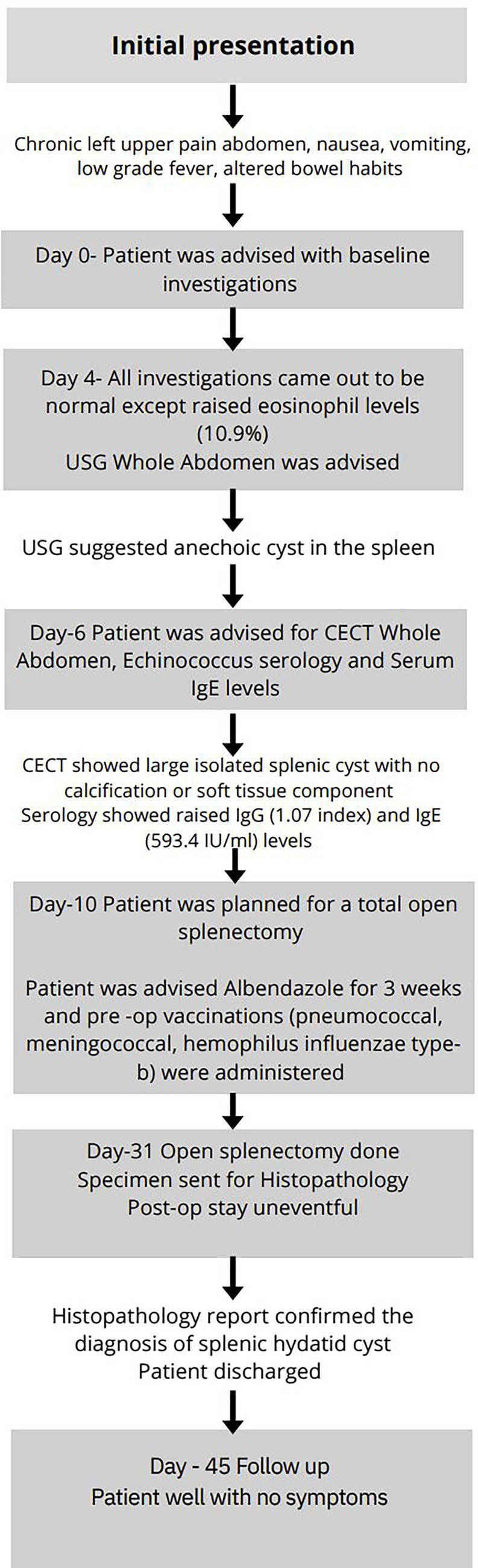
Flowchart depicting the diagnostic timeline since initial presentation.

Following the completion of the preoperative course, the patient was admitted for surgery. A left subcostal incision was made, and the lesser sac was accessed. Intraoperative findings revealed an enlarged spleen with a large cyst extending from the upper to the lower pole. The gastrosplenic and splenocolic ligaments were divided and ligated, followed by division and ligation of the splenorenal and splenophrenic ligaments. Finally, the splenic artery and vein were ligated near the hilum. No scolicidal agent was used intraoperatively, and the spleen was removed en masse. The specimen was sent for histopathological analysis after the splenic cyst was drained (Figure 3). The postoperative period was uneventful, and albendazole was discontinued. At the two-week follow-up, the patient was clinically stable and reported no complaints and the wound site appeared healthy. A long-term follow-up plan was established, with periodic ultrasonography scheduled at 3, 6, and 12 months after surgery. The comprehensive timeline, from initial presentation to surgical intervention and follow-up, is illustrated in Figure 4.

The histopathological examination confirmed the diagnosis of splenic hydatid disease. The gross specimen measured 13.5 × 9 × 4.5 cm, with the external surface showing signs of congestion. Serial sectioning revealed a cystic cavity measuring 8 × 7 × 4 cm, with a cyst wall thickness of 0.3 cm. Within the specimen container, two pearly white, glistening, flap-like tissue fragments were identified, one measuring 10 × 7 cm and the other 14 × 5 cm. Microscopic analysis demonstrated that the cyst wall consisted of an outer acellular laminated membrane, adjacent to a dense fibrotic pseudocapsule layer containing granulation tissue with a marked mixed inflammatory infiltrate. This infiltrate included eosinophils, neutrophils, lymphocytes, plasma cells, macrophages, and multinucleated giant cells, along with numerous blood vessels of varying thickness. A transparent germinal membrane was observed, although no protoscolices were present. The attached splenic tissue showed prominent lymphoid follicles with well-formed germinal centers and intervening sinusoids. The splenic hilum and overlying capsule appeared unremarkable, and no atypical cells were noted. The cyst wall tested positive for periodic acid-Schiff (PAS) stain.

## Discussion

Our management strategy had both strengths and limitations. A major strength was the timely diagnosis, achieved through strong clinical suspicion and supportive imaging findings. According to the WHO-IWGE guidelines, our case fulfilled the criteria for possible preoperative diagnosis (11). Given the high risk of rupture, we performed prompt surgical intervention with histopathological confirmation. Total open splenectomy was selected because of the cyst’s large size, friability, and the absence of infection or malignancy. Although laparoscopic splenectomy is increasingly reported, the cyst’s characteristics warranted an open approach.

However, our approach had limitations, notably the absence of standardized consensus regarding optimal management, the lack of long-term follow-up, and the omission of scolicidal agents. Given the patient’s young age, surveillance is essential to identify potential complications such as cyst recurrence or the development of extrasplenic cysts, which could significantly impact her quality of life. While total splenectomy is recommended for large or symptomatic cysts, alternative approaches, including spleen-preserving surgery and adjunctive medical therapy, have also been described. However, recent evidence indicates that spleen-preserving techniques for large or multiple cysts carry higher risks of hemorrhage, residual cavity infection, and prolonged recovery, which frequently outweigh their benefits (4, 6). Although the omission of scolicidal agents may increase the risk of recurrence and secondary CE, their routine use remains controversial due to potential complications such as sclerosing cholangitis, cytotoxicity, non-standardized preparation, and primarily parasitostatic effects (12). Similar to recent cases, swift diagnosis and surgical intervention proved critical for our patient, reinforcing the necessity of maintaining high suspicion for isolated splenic hydatid cysts even in atypical settings, as diagnostic delays significantly worsen outcomes (13).

Patients with splenic hydatid disease present with varied clinical features. While some patients may be completely asymptomatic (14), others typically experience dull, dragging pain as the initial symptom, along with dyspepsia, constipation from colonic pressure, or dyspnea due to left diaphragm elevation (5). In many cases, an incidentally discovered mass in the left hypochondrium serves as the first clinical indicator of a splenic hydatid cyst (5). Diagnosis is often challenging, as hydatid cysts may mimic simple cysts and lack typical serological or imaging features, closely resembling other splenic lesions such as epidermoid cysts, pseudocysts, hematomas, abscesses, or cystic neoplasms (5, 13). If left undiagnosed, progressive enlargement of the splenic cyst can result in atrophy of the splenic parenchyma and perisplenic inflammation. These pathological processes can lead to adhesions that may develop into fistulas communicating with adjacent structures, including the stomach, pancreatic tissue, descending colon, left kidney, or bronchopulmonary tree (8). Other reported complications include secondary infection of the cyst, traumatic or spontaneous rupture, and portal hypertension secondary to compression of the splenic vein (13, 15). Given these risks, maintaining a high index of suspicion for additional intra-abdominal hydatid cysts is critical to ensure comprehensive evaluation and reduce the likelihood of future morbidity (16).

Various imaging modalities, including USG, CT, and MRI, can be used to evaluate splenic cystic lesions. USG is typically the first-line imaging modality, as it can identify hydatid sand, daughter cysts, and membranes, and plays a central role in the screening, follow-up, and interventional management of abdominal hydatid disease. Non-contrast CT is beneficial for detecting calcifications, for assessing obese patients, or when USG findings are inconclusive. It is regarded as the most sensitive modality for evaluating SHCs, including their number, size, and location (17). MRI is generally reserved for patients with negative serology or inconclusive USG/CT findings. It is superior in demonstrating cyst wall defects, biliary communication, and neural involvement and in differentiating hydatid cysts from simple cysts, although its higher cost limits routine use (1, 8, 17). Chest radiographs remain the most reliable test for pulmonary hydatid cysts, with a diagnostic accuracy of 99%, while chest CT may be used as an additional modality but is not essential (18). When combined with serological tests such as ELISA, immunoelectrophoresis, or indirect hemagglutination, imaging can accurately diagnose splenic hydatid disease in up to 90% of patients (4).

Surgery is often the most commonly selected treatment modality, and the type of surgery is based on individual factors. Options include total splenectomy or conservative procedures such as partial splenectomy, cyst enucleation, or deroofing of the cyst with omentoplasty or external drainage (5, 6). Total splenectomy is especially recommended for cases involving large cysts occupying more than 75% of the splenic parenchyma, cysts with adhesions or invasion into adjacent organs, multiple symptomatic cysts, or cysts located at the splenic hilum (6). However, it is associated with an increased risk of OPSI, bleeding, thromboembolic events (occurring in 6–11% of patients), and intra-abdominal abscesses (10). For small, solitary, peripherally located (polar) cysts, conservative surgery is often preferred (1, 6). These techniques reduce the risk of OPSI but may be complicated by hemorrhage, residual cavity infection, or recurrence (5, 6, 8). Nonetheless, some studies report no significant difference in recurrence rates between conservative procedures and total splenectomy (6). Given these considerations, intraoperative frozen section analysis, when combined with preoperative imaging, can guide surgical decision-making and support splenic preservation, which is especially important in pediatric patients (19).

With regard to the surgical approach, both open and laparoscopic techniques have been described, each offering advantages and limitations. The laparoscopic approach is associated with a higher risk of cyst rupture, peritoneal spillage, and anaphylaxis and is unsuitable for larger cysts (8, 9). However, it offers certain benefits, including reduced postoperative pain, shorter hospital stays, and a decreased risk of surgical site infections and complications associated with prolonged bed rest (6). With advances in minimally invasive techniques, laparoscopic partial splenectomy is being increasingly preferred, as it preserves splenic function, demonstrates recurrence rates of <2% compared to 50% after total splenectomy, has minimal complications with <1% risk of incisional hernia, and rarely requires conversion to open surgery or transfusion (14). The choice of approach should be individualized based on cyst size, location, patient comorbidities, and surgical expertise, with splenic preservation particularly emphasized in pediatric patients.

A newer minimally invasive treatment option is the Puncture, Aspiration, Injection, Re-aspiration (PAIR) technique. It is considered most suitable for WHO-IWGE Type I and Type II cysts, particularly those measuring less than 5 cm in diameter, and for patients who are poor candidates for surgery (9).

Anthelmintic therapy has a special role in the treatment of hydatid disease. Preoperative albendazole facilitates the removal of the germinal layer and sterilizes the cyst (6, 13). It also lowers intracystic pressure and decreases the risk of anaphylaxis during surgical manipulation (6, 9). Postoperative anthelmintic therapy is not necessary in cases of isolated splenic cyst resection or successful total splenectomy, but it is recommended following spleen-sparing surgery, recurrence, or extra-splenic disease. The duration of therapy ranges from 2 to 6 weeks following splenectomy and from 3 to 8 weeks after PAIR (9). Albendazole must be used cautiously in patients with chronic liver disease and avoided in those with bone marrow suppression. It is not indicated for inactive or calcified cysts, and its effect is noticeably slower in cysts larger than 10 cm due to the large volume of fluid (11).

Despite these options, no standardized management protocols exist for cysts with intermediate size (5–10 cm), atypical imaging features, or discordant clinical findings. Prospective studies directly comparing surgical strategies and long-term outcomes, such as recurrence and OPSI incidence, are needed to establish evidence-based treatment algorithms.

## Conclusion

Splenic echinococcosis is often overlooked in patients presenting with vague abdominal symptoms, leading to delayed diagnosis and poor outcomes. When detected through appropriate imaging and serological tests, the prognosis is generally favorable. Surgical removal of the cyst, with or without total splenectomy, remains the primary mode of treatment. Preoperative administration of albendazole, along with vaccination against *Streptococcus pneumoniae, Neisseria meningitidis, and Haemophilus influenzae* type b, is recommended to prevent postoperative complications, particularly in cases requiring splenectomy. This case highlights the importance of prompt recognition and intervention, as improved clinical awareness can enable earlier diagnosis and improve patient outcomes.

## Data Availability

The original contributions presented in the study are included in the article/supplementary material, further inquiries can be directed to the corresponding author.
